# MicroRNA-1246 Mediates Drug Resistance and Metastasis in Breast Cancer by Targeting NFE2L3

**DOI:** 10.3389/fonc.2021.677168

**Published:** 2021-12-01

**Authors:** Yue-chu Dai, Yin Pan, Ming-ming Quan, Qi Chen, Yue Pan, Yan-yun Ruan, Jian-guo Sun

**Affiliations:** ^1^ Department of Surgical Oncology, Taizhou Central Hospital (Taizhou University Hospital), Taizhou, China; ^2^ Precision Medicine Center, Taizhou Central Hospital (Taizhou University Hospital), Taizhou, China

**Keywords:** breast cancer, miR-1246, drug resistance, metastasis, NFE2L3, epithelial-to-mesenchymal transition 3

## Abstract

MicroRNA (miR)-1246 is abnormally expressed and has pro-oncogenic functions in multiple types of cancer. In the present study, its functions in breast cancer and the underlying mechanisms were further elucidated. The clinical relevance of miR-1246 was analyzed and its expression in clinical specimens and cell lines was examined by reverse transcription-quantitat000000ive PCR analysis. FACS was used to detect cell apoptosis and mitochondrial transmembrane potential. A Transwell system was used to detect cell migration and invasion. Luciferase assay was used to confirm the target gene of miR-1246. Xenograft and metastasis mouse models were constructed to determine the function of miR-1246 *in vivo*. miR-1246 was found to be negatively associated with overall survival in breast cancer. miR-1246 inhibitor could effectively increase the cytotoxicity of docetaxel (Doc) by inducing apoptosis, and impair cell migration and invasion by suppressing epithelial-to-mesenchymal transition. Nuclear factor (erythroid 2)-like factor 3 (NFE2L3) was confirmed as a new target gene of miR-1246, and its overexpression was shown to reduce drug resistance and migration of MDA-MB-231 cells. More importantly, NFE2L3-silencing attenuated the effect of miR-1246 inhibitor. Finally, the inhibition of miR-1246 effectively enhanced the cytotoxicity of Doc in xenografts and impaired breast cancer metastasis. Therefore, miR-1246 may promote drug resistance and metastasis in breast cancer by targeting NFE2L3.

## Introduction

Breast cancer is the second most common cause of cancer mortality among women (1). Chemotherapy is a critical therapeutic approach for breast cancer, and various chemotherapeutic agents are used for this purpose. However, almost all patients with breast cancer can develop drug resistance, which comprises one of the most serious challenges in cancer treatment (2). It is believed that the overall survival of cancer patients could be effectively improved if drug resistance could be overcome (3). Furthermore, metastasis, another unique characteristic of cancer progression, is the primary cause of breast cancer morbidity and mortality (4). Therefore, elucidating the molecular mechanisms underlying metastasis and chemotherapy resistance is urgently required to develop more effective therapeutic strategies and agents for the treatment of patients with late-stage breast cancer.

Recently, an increasing number of studies have focused on non-coding RNAs, such as microRNAs (miRNAs) and long non-coding RNAs (lncRNAs). miRNAs are small non-coding RNAs containing ~22 nucleotides, which can negatively regulate the expression of genes by binding to the 3’-untranslated region (3’-UTR) of their target mRNAs (5). Accumulating evidences have indicated that miRNAs played important roles in the regulation of cell proliferation, apoptosis, migration, invasion, and metabolism (6). An increasing number of miRNAs has been reported to be abnormally expressed in human breast cancer. miRNAs are also intricately involved in the development and progression of breast cancer. For example, miR-346 may promote docetaxel (Doc) resistance in breast cancer cells by downregulating SRCIN1 (7), and miR-3646 causes Doc resistance in human breast cancer cells *via* the Wnt signaling pathway (8). The expression of miR-34c has been reported to be significantly suppressed in the metastatic lesions of breast cancer, and its mimics may inhibit cancer cell migration and invasion by impairing GIT1 expression (9). Recently, miR-125a-5p was found to be significantly reduced in human breast cancer specimens, and may exhibit an anticancer function during carcinogenesis by targeting breast cancer susceptibility gene 1-associated protein 1 translation (10).

Our previous study revealed that an elevated miR-1246 levels were detected in the serum of patients with breast cancer, as compared with those in healthy controls (11). Recently, increasing evidences prove miR-1246 plays important oncogenic roles in multiple cancer types such as colorectal cancer (12, 13), and lung cancer (14). However, its biological function and the underlying mechanism in breast cancer remain poorly understood. In the present study, the pro-oncogenic effects of miR-1246 in human breast cancer cells and underlying mechanism were investigated. The expression of miR-1246 in breast cancer tissues and whether its functions on drug resistance and migration ability of human breast cancer cells *in vitro* and *in vivo* was examined. It was also investigated whether nuclear factor (erythroid 2)-like factor 3 (NFE2L3) is a novel target gene of miR-1246 in human breast cancer cells, and whether its overexpression or silence could affect Doc resistance, cell migration and invasion of breast cancer cells, and the role of miR-1246. These findings may uncover whether targeting miR-1246/NFE2L3 axis could be a potential strategy for overcoming drug resistance and metastasis in human breast cancer.

## Materials and Methods

### Cell Culture and Clinical Specimens

The MCF-7, MDA-MB-231, MDA-MB-468 and SKBR3 human breast cancer cell lines were obtained from the American Type Culture Collection and cultured in DMEM (Thermo Fisher Scientific, Inc.) supplemented with 10% fetal bovine serum (FBS; Cytiva), 100 U/ml penicillin and 100 g/ml streptomycin. All cell lines were cultured in a humidified incubator in an atmosphere of 5% (v/v) CO_2_ at 37°C.

Paired primary breast cancer specimens and paracancerous tissues were collected from 20 patients who underwent surgery at the Taizhou Central Hospital (Taizhou, China) between September 2016 and March 2017, and immediately stored in liquid nitrogen until further use. Written informed consent for the use of these clinical materials in the present study were provided by all patients, and the study protocol was approved by the Institutional Ethics Committee of the Taizhou Central Hospital.

### Chemicals and Antibodies

Antibodies against caspase-3 (#56053) and compound ABT-737 were purchased from Santa Cruz Biotechnology, Inc.; antibodies against AKT (#4691), p-AKT (#4060), ERK (#4695), p-ERK (#4370), NF-κB (#8242), p-NF-κB (#3033), E-cadherin (#3195), vimentin (#5741), N-cadherin (#4068), caspase-8 (#4790), caspase-9 (#9504) and IAP family proteinsc-IAP1 (#7065), c-IAP2 (#3130), survivin (#2808), XIAP (#2045) and livin (#5471) were obtained from Cell Signaling Technology, Inc. Antibodies against β-actin (#A2228) and NFE2L3(#HPA055889) were obtained from Merck KGaA. Antibodies against Bcl-2 (#32124), Mcl-1 (#32087), Bad (#32245), and Bak (#32371) were purchased from Abcam. All antibodies were diluted at 1:1000. Annexin V-FITC was purchased from BD Biosciences. JC-1 was obtained from ThermoFisher Scientific, Inc. Doc was purchased from Selleck Chemicals. All other reagents were from Sigma-Aldrich; Merck KGaA. All drugs were dissolved in DMSO and stocked at -80°C.

### Plasmid Construction and Transfection

The coding sequence of human NFE2L3 mRNA was synthesized, digested, and linked into the overexpression pcDNA3.1 vector. The integrity of the respective plasmid constructs was confirmed by DNA sequencing. After cells were seeded in 6cm dish overnight, 2 μg pcDNA3.1-NFE2L3 plasmid or pcDNA3.1 vector was transfected using Lipofectamine^®^ 3000 (Thermo Fisher Scientific, Inc.), according to the manufacturer’s instructions. Western blotting was used to determine the efficiency of pcDNA3.1-NFE2L3 plasmid transfection.

The potential binding site of miR-1246 on NFE2L3 mRNA 3’UTR was predicted by the databases of TargetScan, ENCORI, and Mirtarbase, and the 200 bp fragments up- and downstream of the binding site were synthesized to construct the PGL3-NFE2L3 wild-type (WT) and PGL3-NFE2L3 mutant (MUT) plasmids for 3’UTR reporter assays. Briefly, 1 μg PGL3-NFE2L3 WT and PGL3-NFE2L3 MUT plasmids were used to perform the transfection with Lipofectamine^®^ 3000, and PGL3 empty vector was used as a negative control (NC). PRL-CMV plasmid (24 ng) encoding Renilla luciferase was included in all transfections to normalize transfection efficiency.

### Oligonucleotide Transfection

Inhibitor and mimics for miR1246, as well as their NC, were purchased from Shanghai GenePharma Co., Ltd. Following seeding in a 6-well plate overnight, the cells were transfected with miR1246 inhibitor (mimics) or NC inhibitor (mimics; 50 nM) using Lipofectamine^®^ 3000.

The siRNA sequence against NFE2L3 was 5’-GCACGAAGCUGUGGAUAUTT-3’ and was obtained from Genepharma. siRNA (100 nmol) was used to perform the silencing experiment using Lipofectamine^®^ 3000, and NC siRNA was used as the control. Western blotting was used to determine the silencing efficiency.

### MTT Assay

Following seeding in 96-well plates overnight, cells were incubated with various concentrations of anticancer agents for 24 h, and then the medium was discarded. Next, 50 μl of 1 mg/ml MTT was added to each well and incubated at 37°C for up to 4 h. The purple formazan formed was then solubilized by DMSO and a microplate reader (Molecular Devices, LLC) was used to detect the absorbance at 570 nm.

### Apoptosis Analysis

Following treatment, cell apoptosis was analyzed by flow cytometry (FASCanto; BD Biosciences) by staining with FITC-labeled Annexin V and propidium iodide (PI). MinWID 2.9 (BD Biosciences) was used for data acquisition and analysis. The summation of both early (Annexin V^+^ and PI^-^) and late (Annexin-V^+^ and PI^+^) apoptotic cells was used to determine the percentage of cells undergoing apoptotic death.

### Measurement of Mitochondrial Transmembrane Potential (ΔΨm)

The fluorescent cationic dye JC-1 was used to analyzed the ΔΨm. Following washing with PBS, cells were resuspended in 500 μl PBS and stained with 10 μM JC-1 at 37°C in the dark for 15 min. Flow cytometry was then performed. MinWID 2.9 (BD Biosciences) was used for data acquisition and analysis.

### Transwell Assays for Cell Migration and Invasion

Cell migration was determined using a Transwell system (8-μM pore; Corning Life Sciences) according to the manufacturer’s instructions. Following suspension in serum-free DMEM, cells were seeded into the upper Transwell chambers. The lower compartment was placed into 24-well plates and filled with DMEM with 10% FBS as a chemoattractant. The cells located in the upper chamber were removed with a cotton swab after 24 h incubation. Following fixing with methanol and staining with 0.5% crystal violet solution for 30 min at room temperature, a microscope (CKX53, Olympus Corporation) was used to count the number of cells on the lower surface of the polycarbonate membrane at 200X magnification. Data from three independent experiments were used to determine the mean number of migrated cells. Cell invasion potential was determined using a Matrigel-coated transwell chamber followed the same protocol as cell migration detection assay.

### Western Blot Analysis

Lysis buffer containing 2.1 μg/ml aprotinin, 0.5 μg/ml leupeptin, 4.9 mM MgCl2, 1 mM orthovanadate, 1% Triton X 100 and 1 mM PMSF was used for protein extraction from the cells. 20 μg protein was uploaded for each sample and separated with 12% denaturing SDS-PAGE and transferred to a PVDF membrane (EMD Millipore). The membranes were first blocked with 5% fat-free dry milk for 2 hours at room temperature, and then washed with PBS containing 0.1% Tween-20. Subsequently, the membrane was incubated with primary antibodies and respective secondary antibodies. Enhanced chemiluminescence detection reagents (GE Healthcare) and X-ray film (Fujifilm) were used to visualized the signal. β-Actin was used as the loading control.

### Reverse Transcription-Quantitative (RT-qPCR) Analysis

Total RNA was extracted by TRIzol^®^ reagent (Thermo Fisher Scientific, Inc.) and then used to synthesize the first-strand cDNA by M-MLV reverse transcriptase (Promega Corporation). qPCR amplification was performed in an ABI 7300 Real-Time PCR system (Thermo Fisher Scientific, Inc.), according to the manufacturer’s instructions for relative quantification. 2X Power SYBR Green PCR Master Mix (Thermo Fisher Scientific, Inc.) was used to carry out the amplification reactions. The standard RT-qPCR protocol included initial denaturation at 95°C for 10 min, followed by 40 cycles of denaturation at 95°C for 15 sec, annealing and extension at 60°C for 1 min. A DNA dissociation curve was generated to confirm the specificity of the amplification. The relative mRNA expression was determined by the 2^-ΔΔCq^ method using β-actin or U6 as controls.

The primer sequences for RT-qPCR were as follows: miR-1246, 5’-GTCGTATCCAGTGCAGGGTCCGAGGTATTCGCACTGGATACGACCCTGC-3’ reverse transcription, 5’-AATGGATTTTTGG-3’ forward and 5’-CACTGGATACGAC-3’ reverse; NFE2L3, 5’-GGGAAAAATAAAGTTGCTGCG-3’ forward and 5’-GGTTGGGATTGACTGGCCTA-3’ reverse; E-cadherin, 5’-GCTCACATTTCCCAA CTC-3’ forward and 5’-GTGGCAATGCGTTCTCTA-3’ reverse; N-cadherin, 5’-GCACCCCTTCACCCAACA-3’ forward and 5’-GGCGAACCGTCCAGTAGG-3’ reverse; vimentin, 5’-TGCGTGAAATGGAAGAGAACTT-3’ forward and 5’-TGGGTATCAACCAGAGGGAGTG-3’ reverse; β-actin, 5’-AGCACAGAGCCTCG CCTTTGC-3’ forward and 5’-CTGTAGCCGCGCTCGGTGAG-3’ reverse; U6, 5’-CTCGCTTCGGCAGCACA-3’ forward and 5’-AACGCTTCACGAATTTGCGT-3’ reverse.

### Luciferase Reporter Activity Assay

After MDA-MB-231 cells were seeded in 24 well plate for 24 h, cells were transfected with 1.2 μg luciferase-reporter PGL3-NFE2L3 WT or PGL3-NFE2L3 MUT plasmids using Lipofectamine^®^ 3000 (Thermo Fisher Scientific, Inc.), and PGL3 empty vector was used as a NC. pRL-CMV plasmid (24 ng) was co-transfected to normalize transfection efficiency. After 24 h incubation, cells were washed with PBS and lysed with the passive lysis buffer from the Dual-Luciferase Reporter Assay System kit (Promega Corporation). Luciferase assay was performed using a FLUOstar Galaxy plate reader (Promega Corporation).

### Assessment of Reactive Oxygen Species

Cell were harvested and single cell suspension was prepared by gently pipetting up and down. Cells were stained in culture media with 20 μM DCFDA for 30 minutes at 37°C. After staining, cells were analyzed on flow cytometer, DCFDA should be excited by the 488 nm laser and detected at 535 nm (typically FL1).

### Xenograft Experiment

MDA-MB-231 cell-derived xenografts were established by injecting 5x10^6^ cells subcutaneously into nude mice. After the solid tumor grew to ~100 mm^3^, the tumor-bearing mice were randomized into two groups, each with 5 mice. The mice in the treatment group were injected intravenously with miR-1246 antagomir every 2 days (50 nM for each mouse, obtained from Shanghai GenePharma Co.). The control mice were given NC antagomir intravenously at the same dose. Both groups were given Doc (30 mg/kg) intravenously twice every week. A micrometer caliper was used to measure the 3 diameters of the tumor every 2 days to calculate the tumor volume. Data are expressed as the mean ± SEM.

### Experimental Lung Metastasis Mouse Model

The experimental lung metastasis mouse model was established as previously described (15). All animal experiments in the present study were approved by the Animal Experimentation Ethics Committee of Taizhou University (Taizhou, China).

### Immunohistochemistry and TUNEL Staining

Following the completion of all treatments, mice were sacrificed by cervical dislocation and their tumors were removed before their volume had exceeded 2,000 mm^3^. Tumors were fixed using 10% formaldehyde overnight at room temperature. Following dehydration with various concentrations of ethanol and washing with xylene, tumors were embedded in paraffin and cut into 5-μm sections. Subsequently, sections were subjected to immunohistochemistry to detect the NFE2L3 expression following deparaffinization with xylene, washing and rehydration in graded ethanol. A commercially available kit (TUNEL Assay Kit - BrdU-Red, Abcam) was used to perform the TUNEL assay, according to the manufacturer’s instructions. Apoptotic cells were stained red. DAPI was used as a counterstain.

### Dataset and Statistical Analysis

Survival analysis was performed for miRNAs (excluding putative miRNAs) using the METABRIC miRNA-expression dataset. Kaplan-Meier analyses (www.KMplot.com) was applied to obtain survival curves and log-rank test was applied to evaluate the significance of group differences in survival rates.

All results are expressed as the mean ± standard deviation from at least three independent experiments. GraphPad Prism 5.0 (GraphPad Software, Inc.) was used to perform all statistical analyses. Statistical significance was assessed using a two-sided Student’s t-test for datasets containing two groups, or two-way ANOVA (parametric) with a Bonferroni post-test for the comparison of multiple groups, and P<0.05 was considered to indicate a statistically significant difference.

## Results

### MiR-1246 Is Negatively Correlated With Overall Survival in Patients With Breast Cancer

**Figure 1:**
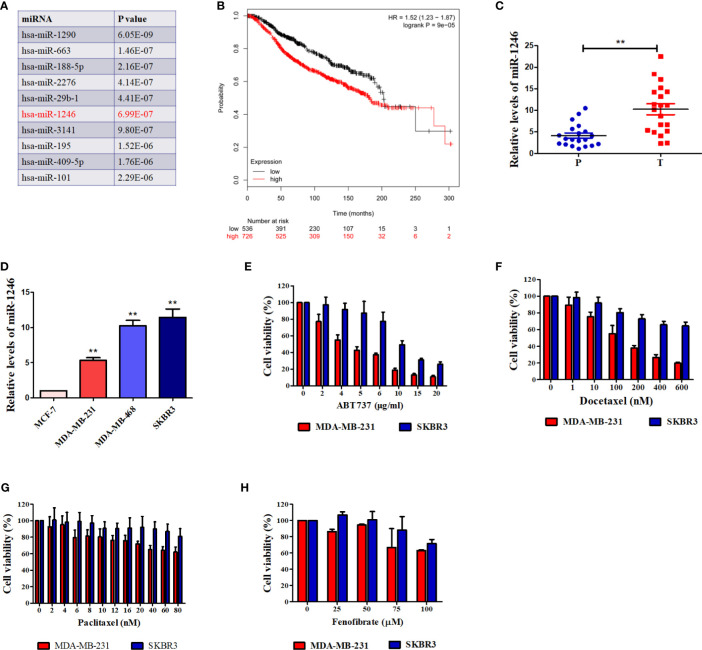
miR-1246 is correlated with survival of breast cancer patients and drug resistance of breast cancer cells. **(A)** Top 10 miRNAs were listed according to their significance of correlating with overall breast cancer patient survival. **(B)** Correlation between expression of miR-1246 and overall survival of breast cancer patients. Data were obtained from the Kaplan Meier Plotter. **(C)** miR-1246 levels in 20 paired human breast cancer tissues and paracancerous tissues were determined by RT-qPCR. P, paracancerous tissues; C, breast cancer tissues. **(D)** miR-1246 levels expressed in human breast cancer cell lines were examined by RT-qPCR. (E to H) The cytotoxicity of ABT-737 **(E)**, Paclitaxel **(F)**, Docetaxel **(G)** and Fenofibrate **(H)** in the indicated cells were determined by MTT analysis. Data are presented as mean ± SD, n=3. **P < 0.01 vs. control.

Our previous study revealed that serum miR-1246 levels in patients with breast cancer were elevated, as compared with those in healthy controls (11). An increasing number of studies have indicated that miR-1246 exerted pro-oncogenic activity in various types of human cancer (16–18). Bott et al. analyzed the data from a METABRIC miRNA-expression dataset (19) to screen the miRNAs those were significantly correlated with the overall survival of patients with breast cancer (18). The top 10 miRNAs correlated with breast cancer patient survival are listed in **Figure 1A**, with miR-1246 ranking 6th (P<0.01). We also analyzed the clinical relevance of miR-1246 in patients with breast cancer in a public dataset (www.KMplot.com), and a negative association between miR-1246 levels and overall survival was observed (log-rank P<0.01; **Figure 1B**). Finally, miR-1246 levels in 20 pairs of breast cancer tissues and corresponding paracancerous tissues were compared using RT-qPCR analysis, and the data indicated that the expression level of miR-1246 was significantly elevated in tumor specimens (P<0.01; **Figure 1C**).

### MiR-1246 Promotes Resistance of Breast Cancer Cells to Anticancer Agents

The genotype of estrogen receptor (ER) is a critical predictor of overall survival in breast cancer (20). Therefore, the miR-1246 levels in 4 human breast cancer cell lines with different ER genotypes were detected. As shown in **Figure 1D**, the levels of miR-1246 were higher in ER^-^ (MDA-MB-231, MDA-MB-468 and SKBR3) compared with that in ER^+^ (MCF-7) breast cancer cells. MTT assay was used to confirm the cytotoxicity of different anticancer drugs in two ER-negative breast cancer cell lines (MDA-MB-231 and SKBR3). It appeared that cells with a higher miR-1246 level exhibited stronger resistance to multiple antitumor drugs, including ABT-737 (**Figure 1E**), paclitaxel (**Figure 1F**), Doc (**Figure 1G**) and fenofibrate (**Figure 1H**).

### MiR-1246 Inhibition May Reverse Drug Resistance in Human Breast Cancer Cells by Inducing Apoptosis

**Figure 2:**
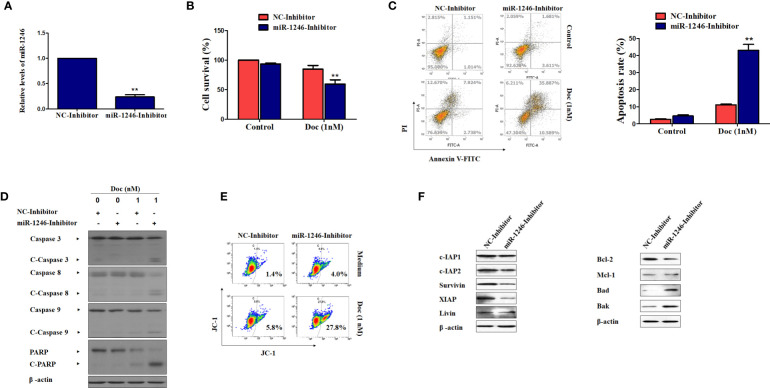
miR-1246 inhibition effectively increase the sensitivity of MDA-MB-231 cells to docetaxel by inducing apoptosis. **(A)** MDA-MB-231 cells were transfected with specific inhibitor against miR-1246 or negative control (NC) and the silence efficiency was confirmed by RT-PCR. **(B)** The cytotoxicity of 1nM DOC in MDA-MBA-231 cells transfected with miR-1246 inhibitor or NC were determined by MTT assay. **(C)** After transfection of miR-1246 inhibitor or NC, Doc induced apoptosis in MDA-MB-231 cells were detected by flow cytometry. **(D)** The cleavage of caspase 3, 8, 9 and PARP were also determined by western blotting in MDA-MB-231 cells treated with Doc in combination with miR-1246 inhibitor or NC. **(E)** The mitochondrial membrane permeability was tested by flow cytometry after JC-1 staining in cells from **(D)**. **(F)** The IAPs and Bcl-2 family members expression was determined by western blot assay in MDA-MB-231 cells transfected with miR-1246 inhibitor or NC. Data are presented as mean ± SD, n=3. **P < 0.01 vs. control.

To further validate the function of miR-1246 in the drug resistance of human breast cancer cells, inhibitor against miR-1246 and NC were synthesized. After MDA-MB-231 cells were transfected with the miR-1246 or NC inhibitor for 24 h, the inhibitory effect was determined by RT-qPCR analysis (**Figure 2A**). MTT assay was performed to determine whether miR-1246 inhibition could increase the Doc sensitivity of MDA-MB-231 cells. As expected, the same dose of Doc (1 nM) was more cytotoxic to cells transfected with miR-1246 inhibitor compared with the NC group cells (**Figure 2B**). Next, the flow cytometry data demonstrated that miR-1246 suppression significantly increased Doc-induced apoptosis in MDA-MB-231 cells (**Figure 2C**, P<0.01), which was also supported by the increased cleavage of caspase-3 and PARP (**Figure 2D**).

The present data also revealed that caspase-8 and -9 were cleaved more effectively after cells were treated with Doc in combined with miR-1246 inhibition (**Figure 2D**). These findings suggested that both the extrinsic and intrinsic apoptotic pathways were activated, which was also supported by mitochondrial membrane permeability data (**Figure 2E**). Furthermore, the expression of IAP family members, such as c-IAP1, c-IAP2, survivin, XIAP and livin, and Bcl-2 family members, such as Bcl-2, Mcl-1, Bak, and Bad, which play critical roles in cancer cells apoptosis regulation, was examined by western blotting. Of note, as shown in **Figure 2F**, the suppression of miR-1246 effectively downregulated the expression of almost all IAP proteins indicated and Bcl-2, and elevated the expression of Bak and Bad in MDA-MB-231 cells.

### MiR-1246 Inhibition Significantly Impairs Cell Migration and Invasion in Breast Cancer Cells With Epithelial-To-Mesenchymal Transition Reversal

**Figure 3:**
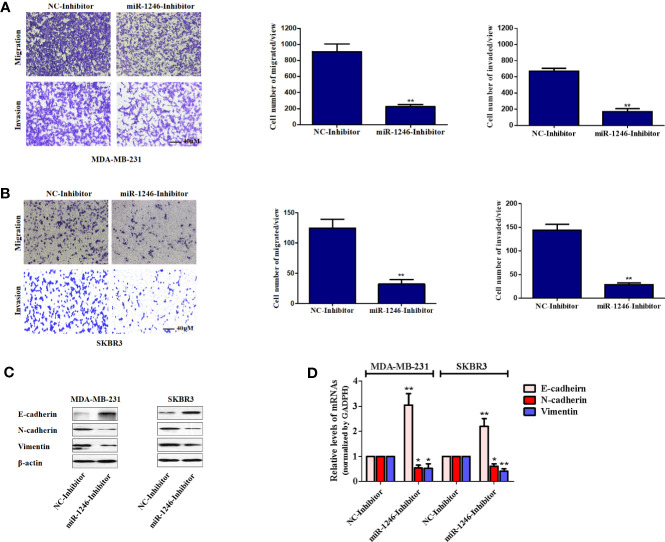
miR-1246 inhibitor impairs the migration and invasion activities of breast cancer cells by repressing EMT. **(A, B)** Migration and invasion abilities of MDA-MB-231 **(A)** and SKBR3 **(B)** cells were determined using a Transwell assay. A total of 2x10^4^ cells were seeded in Transwell chambers and incubated for 24 (h) Magnification: 200X. **(C)** After cells were transfected with miR-1246 inhibitor or NC, the protein levels of EMT biomarkers E-cadherin, N-cadherin and Vimentin were determined using western blotting. **(D)** After cells were transfected with miR-1246 inhibitor or NC, the mRNA levels of EMT biomarkers *E-cadherin*, *N-cadherin* and *Vimentin* were determined using RT-PCR. Data are presented as mean ± SD, n=3. ^*^P < 0.05 vs Control and **P < 0.01 vs. control.

Next, the effect of miR-1246 on the migration and invasion abilities of breast cancer cells was further elucidated. The migration and invasion potentials of both MDA-MB-231 and SKBR3 cells treated with miR-1246 or NC inhibitor was compared by Transwell assay. The present data indicated that miR-1246 inhibitor effectively suppressed the cell migration and invasion of both MDA-MB-231 (P<0.01; **Figure 3A**) and SKBR3 (P<0.01; **Figure 3B**) cells. In addition, the EMT biomarker proteins were measured in these cell lines following miR-1246 inhibitor transfection, as EMT is a critical step for cancer cell metastasis (21). Of note, the inhibition of miR-1246 effectively elevated the expression level of epithelial marker E-cadherin, while suppressing the expression level of mesenchymal markers N-cadherin and vimentin at both the protein (**Figure 3C**) and mRNA (**Figure 3D**) levels. In conclusion, the EMT of MDA-MB-231 and SBBR3 cells was reversed when miR-1246 was inhibited (22).

### NFE2L3 Is a New Target Gene of MiR-1246 in Human Breast Cancer Cells

**Figure 4:**
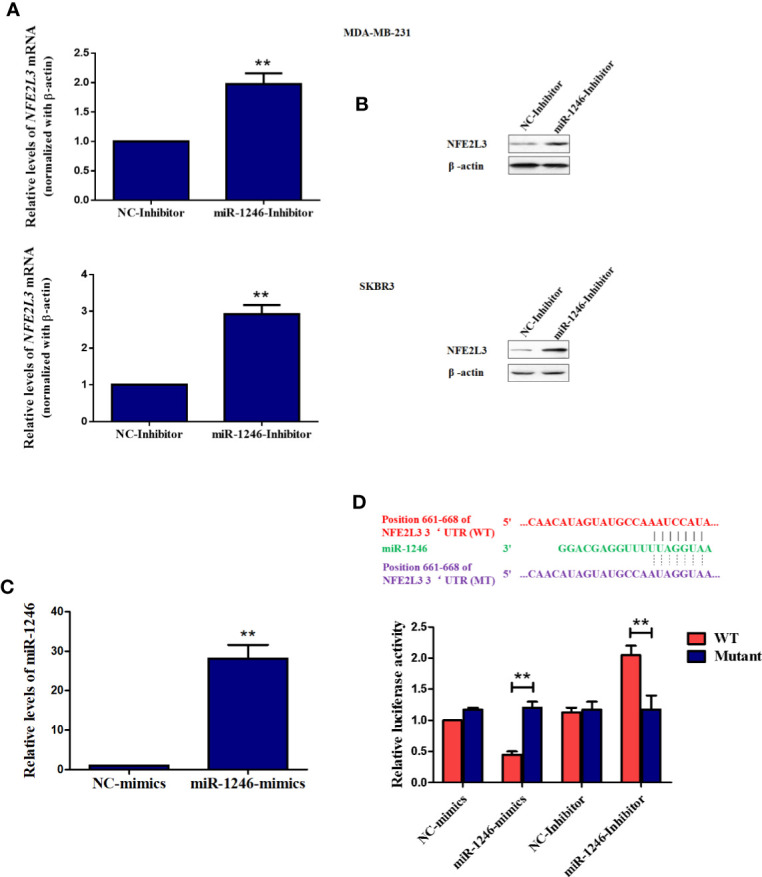
NFE2L3 is a novel target gene of miR-1246 in breast cancer cells. **(A, B)** After MDA-MB-231 and SKBR3 cells were transfected with miR-1246 inhibitor or NC, *NFE2L3* mRNA levels were determined using RT-PCR **(A)** and NFE2L3 protein expression was examined using western blotting **(B, C)** After MDA-MB-231 cells were transfected with miR-1246 mimics or NC, miR-1246 expression levels were determined using RT-PCR. **(D)** 3’UTR assay was performed to confirm the regulatory relationship between miR-1246 and NFE2L3 using wild-type or mutant plasmids. Data are presented as mean ± SD, n=3. **P < 0.01 vs. control.

To further elucidate the mechanisms underlying the biological functions of miR-1246, the datasets of TargetScan, ENCORI, and Mirtarbase was used to screen the potential target mRNA. In this study, NEF2L3 (NRF3) was identified as a new potential target gene of miR-1246. As expected, both the protein and mRNA levels of NFE2L3 were elevated in MDA-MB-231 and SKBR3 cells when miR-1246 was suppressed (**Figures 4A, B**). To further confirm the regulatory association between NFE2L3 and miR-1246, WT and MUT pGL3-NFE2L3 3’UTR plasmids were constructed for 3’UTR reporter assays. The transfection efficiency of miR-1246 mimics was determined using qPCR (**Figure 4C**). The results indicated that miR-1246 mimics inhibited, while miR-1246 inhibitor upregulated the luciferase activity of the WT, but not the MUT pGL3-NFE2L3 3’UTR plasmid (**Figure 4D**). In conclusion, NFE2L3 was confirmed as a direct downstream target gene of miR-1246 in human breast cancer cells.

NFE2L3 belongs to the Cap’n’collar (CNC) protein family, which includes NFE2L1 (NRF1) and NFE2L2 (NRF2) (23). Evidence has emerged supporting that the members of this family play an important role in oxidative stress regulation by promoting the transcription of genes with an antioxidant responsive element in their promoter (24). However, the functions of NFE2L3 in human breast cancer remain poorly understood to date.

### NFE2L3 May Regulate Drug Resistance and Migration in Breast Cancer Cells by Inhibiting Oxidative Stress-Related Downstream Signaling Pathways

**Figure 5:**
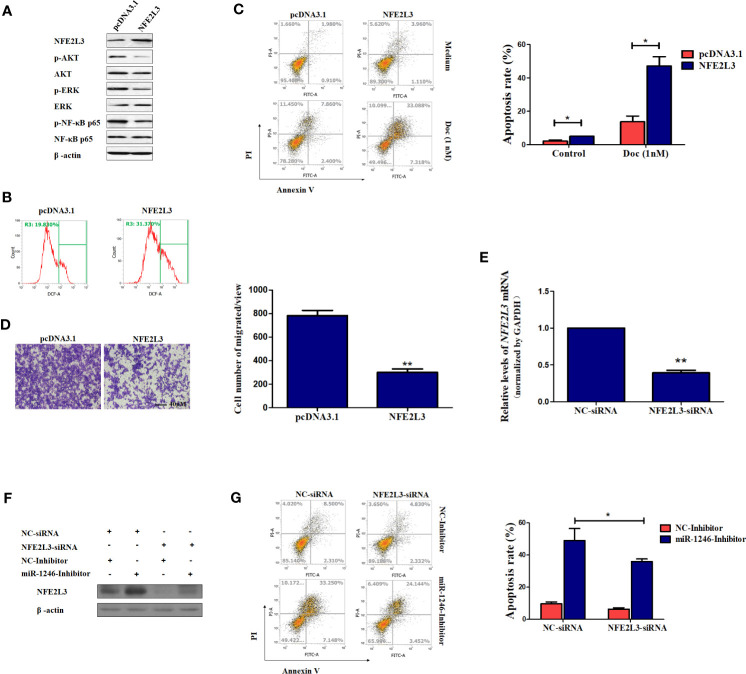
NFE2L3 plays an important role in Doc resistance and migration in MDA-MB-231 cells. **(A)** After cells were transfected with NFE2L3 overexpression plasmid or empty vector for 24h, NFE2L3 expression and the activation of indicated signaling pathways were measured by western blotting. **(B)** Cellular ROS levels were determined by FACS with DCFDA staining in cells from **(A)**. **(C, D)** After cells were transfected with NFE2L3 overexpression plasmid or empty vector for 24h, the Doc induced apoptosis were determined by flow cytometry **(C)** and the migration ability was detected using a Transwell assay, magnification: 200X **(D)**. **(E)** After cells were transfected with NFE2L3 siRNA or NC for 24h, the silence efficiency of NFE2L3 siRNA was determined using qPCR. **(F)** MDA-MB-231 cells were transfected with NFE2L3-siRNA, miR-1246 inhibitor alone or combined. NFE2L3 expression was detected by western blotting. **(G)** After cells were treated as indicated, the Doc induced apoptosis were determined by flow cytometry. Data are presented as mean ± SD, n=3. *P < 0.05 vs. Control and **P < 0.01 vs. control.

It is well known that oxidative stress can promote several aspects of carcinogenesis and tumor progression by activating multiple signaling pathways, including PI3K/AKT, ERK and NF-κB, to promote cellular proliferation, resistance to apoptosis, angiogenesis and metastasis (25). Therefore, the activation of the signaling pathways mentioned above was next detected in MDA-MB-231 cells. As expected, the phosphorylation of AKT, ERK and NF-κB were obviously repressed following NFE2L3 overexpression in MDA-MB-231 cells (**Figure 5A**). However, our data also indicated that the ectopic expression of NFE2L3 effectively promoted the ROS accumulation in MDA-MB-231 cells (**Figure 5B**).

Furthermore, our data also revealed that the ectopic expression of NFE2L3 significantly sensitized the MDA-MB-231 cell to Doc exposure (P<0.01; **Figure 5C**) and impaired cell migration ability of MDA-MB-231 cells (P<0.01; **Figure 5D**). On the other hand, NFE2L3 silencing using siRNA not only decrease the NFF2L3 mRNA expression (P<0.01; **Figure 5E**), but also significantly attenuated the function of the miR-1246 inhibitor, suppressing DOC-induced apoptosis by ~50% (P<0.01; **Figures 5F, G**). In combination, the present data revealed that the miR-1246/NFE2L3 axis critically affected the malignant properties of human breast cancer cells, at least partially through regulating the activation of several signaling pathways.

### Inhibition of MiR-1246 Can Effectively Enhance the Cytotoxicity of Doc in a Breast Cancer Xenograft Model

**Figure 6:**
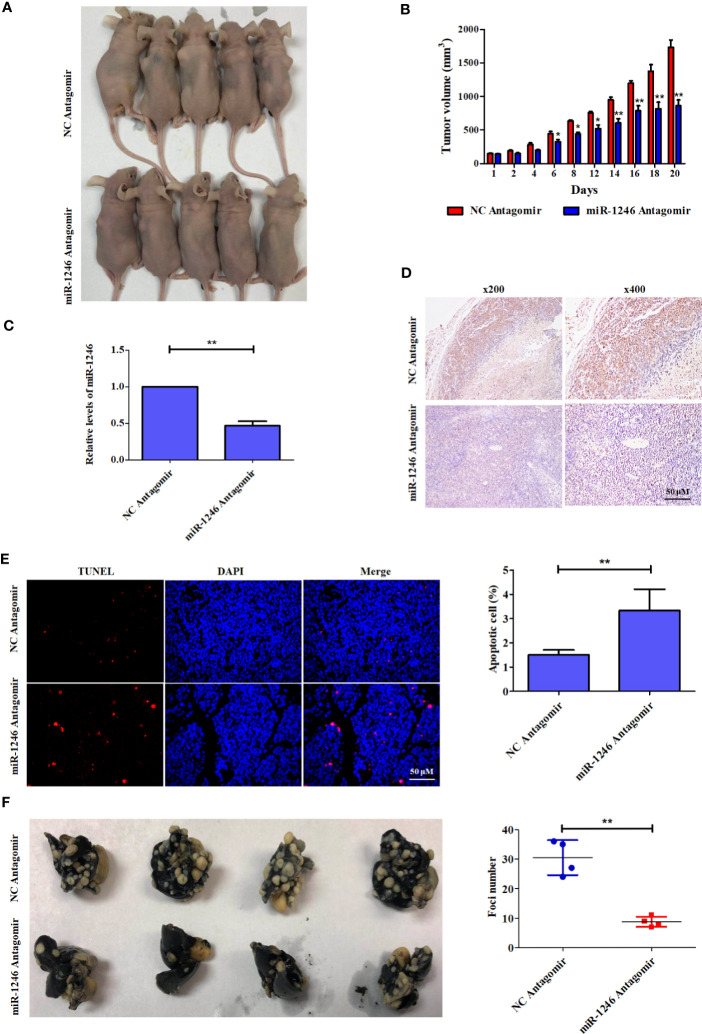
Antagonist of miR-1246 suppressed the drug resistance and metastasis of breast cancer cells in animal model. **(A)** 5x10^6^ MDA-MB-231 cells were subcutaneously inoculated into each female athymic mouse. After tumors formed, the tumor-bearing mice were randomized into four groups, each with five mice. The control mice were treated with 50 nM NC antagomir intravenously every two days for total 20 days. The treatment groups were injected intravenously with 50 nM miR-1246 antagomir for the same frequency and time. All mice were treated with Doc at dose of 30 mg/kg every 2 days for total 20 days. Tumor size was measured in 3 diameters with micrometer caliper every two days to permit calculation of tumor volume. **(B)** Quantitative analysis of tumor size was performed. Data are expressed as the mean ± S.E.M. n=5. *P < 0.05 vs. control. **(C)** The expression of miR-1246 in tumors were measured by qPCR, Data are expressed as the mean ± SD. n=5. *P < 0.05 vs. control. **(D)** IHC assay was used to detect the NFE2L3 expression and the presentative images were shown. **(E)** TUNEL assay was used to detect the cell apoptosis. The presentative pictures were shown (left panel) and quantitative analysis of cell apoptosis was performed (right panel). Data were presented as mean ± S.D, **P < 0.01 versus control group. **(F)** 3x10^6^ MDA-MB-231 cells were injected intravenously to constructed the metastasis mouse model. The control mice were treated with 50 nM NC antagomir intravenously every two days for total 20 days. The treatment groups were injected intravenously with 50 nM miR-1246 antagomir for the same frequency and time. The lungs were obtained and fixed after the mice were sacrificed, and the tumor foci number was counted. The pictures of the lungs were presented (left panel) and quantitative analysis of foci number was performed (right panel). Data were presented as mean ± S.D. N=4. **P < 0.01 versus control group.

To further characterize the pro-oncogenic effect of miR-1246 *in vivo*, the effect of the combination of miR-1246 antagomir and Doc on xenograft tumor growth was analyzed in an MDA-MB-231 cell xenograft model in nude mice. It was indicated that the combined application of miR-1246 antagomir (50 nM) and Doc (30 mg/kg) can inhibit xenograft growth more effectively compared with the combination of NC antagomir and Doc (**Figure 6A**) by ~50% (**Figure 6B**). Meanwhile, miR-1246 expression was inhibited significantly (**Figure 6C**) in tumors of miR-1246 antagomir treated group. Of note, IHC data showed that miR-1246 antagomir significantly upregulated the expression of the NFE2L3 protein in xenografts (**Figure 6D**). The TUNEL assay data also indicated that more cells underwent apoptosis in the group treated with miR-1246 antagomir and Doc, as compared with those in the control (3.36 vs. 1.22%, respectively; **Figure 6E**). In combination, the present data strongly demonstrated that the suppression of miR-1246 can enhance the antitumor effect of Doc, not only *in vitro* but also *in vivo*, likely through the involvement of NFE2L3.

### MiR-1246 Suppression Significantly Impairs the Metastatic Potential of Breast Cancer Cells *In Vivo*

To further determine the pro-metastasis function of miR-1246 *in vivo*, an experimental metastasis mouse model was established. miR-1246 antagomir (50 nM for each mouse) was injected into the lateral tail vein every 2 days, and NC antagomir was used as the control. Of note, the number of the metastatic tumor foci in the lungs of mice treated with miR-1246 antagomir was significantly reduced, as compared those in the NC antagomir control group (~10 vs. 30, respectively; **Figure 6F**). These data also confirmed that the inhibition of miR-1246 could effectively repress the metastasis of breast cancer cells *in vivo*.

## Discussion

Drug resistance and metastasis are the two main challenges in the clinical treatment of human cancer, involving multiple factors and signaling pathways (26, 27). Great efforts have been made to identify the mechanisms underlying drug resistance and metastasis of cancer cells. To the best of our knowledge, the present study was the first report that miR-1246 played important roles in drug resistance and metastasis in human breast cancer by targeting NFE2L3.

Our previous data indicated that miR-1246 levels were elevated in the serum of patients with breast cancer (11). Bott *et al.* analyzed an available public dataset of breast cancer and revealed that miR-1246 is not only strongly associated with poor survival in patients with breast cancer, but also ranks 6th among the most significantly differently expressed miRNAs (**Figure 1A**) (18, 19). This is consistent with the results of the public dataset (www.KMplot.com; **Figure 1B**). Furthermore, the present data indicated that miR-1246 levels were markedly higher in breast cancer tissues and ER-negative breast cancer cell lines, which also supports that this miRNA may serve as a negative predictor of breast cancer prognosis (**Figures 1C, D**).

To further determine the biological functions of miR-1246 in human breast cancer cells, two cell lines MDA-MB-231 and SKBR3 were selected to detect their sensitivity to several anticancer drugs using MTT assay, and the data showed that miR-1246 was positively correlated with the drug resistance of breast cancer cells (**Figures 1E–H**). Next, specific inhibitor against miR-1246 and the NC were synthesized and transfected the MDA-MB-231 cells. The data of MTT and flow cytometry revealed that miR-1246 inhibitor effectively reversed the resistance of MDA-MB-231 cells to Doc by promoting apoptosis (**Figures 2B, C**). Both extrinsic and intrinsic apoptotic pathways were activated, which was supported by the cleavage of caspase-8 and -9, as well as increase of the mitochondrial membrane permeability (**Figures 2D, E**). IAP family members such as c-IAP1, c-IAP2, survivin, XIAP and livin, as well as Bcl-2 family members such as Bcl-2, Mcl-1, Bak, and Bad were detected during miR-1246 inhibition, due to their critical functions in apoptosis regulation. Almost all of anti-apoptotic proteins were downregulated by miR-1246 inhibitor, which was consistent with previous findings indicating that miR-1246 may promote NF-κB signaling pathway activation, as well as that these IAP members and Bcl-2 family were the target genes of the NF-κB transcription factor (**Figure 2F**) (18, 28). Of note, the inhibition of miR-1246 may significantly repress the migration and invasion activities of both MDA-MB-231 and SKBR3 cells, and the level of EMT was also reversed (**Figure 3**).

In the present study, *NFE2L3* was confirmed as a new target gene for miR-1246 by 3’UTR luciferase assay. Both mRNA and protein levels of NFE2L3 were downregulated when the miR-1246 inhibitor was used (**Figures 4A, B**). NFE2L3 is a member of the CNC protein family, which belongs to the basic leucine zipper transcription factors that play a critical role in a number of cellular processes by regulating mammalian gene expression (29). In addition to NFE2L3, vertebrate CNC members also include nuclear factor-erythroid derived 2, NRF1/NFE2L1 and NRF2/NFE2L2, as well as BACH1 and BACH2 proteins with a more distant relationship (30). It is well known that CNC proteins play key roles in oxidative stress response, carcinogenesis and cancer progression (31). Unlike NRF2, little is known on the physiological role of NFE2L3, although several relevant studies have been conducted. In a carcinogenesis-related study, after *Nfe2l3* ^−/−^ mice were treated with the carcinogen benzo[a]pyrene, an increased number of T-cell lymphoblastic lymphomas developed (32). In addition, NFE2L3-deficient mice were not protected from acute lung and adipose tissue damage following treatment with antioxidant agents, such as butylated hydroxytoluene (33). On the other hand, NFE2L3 was shown to activate Pla2g7 expression to promote the differentiation of smooth muscle from stem cells (34). However, the potential roles of NFE2L3 in tumorigenesis and cancer progression remain to be further elucidated.

As mentioned above, oxidative stress is closely associated with carcinogenesis and tumor progression by promoting the activation of multiple downstream signaling pathways, including the PI3K/AKT, ERK and NF-κB pathways (25). Therefore, the phosphorylation of AKT, ERK and NF-κB under NFE2L3 overexpression was next detected. As expected, the phosphorylation of AKT, ERK and NF-κB was effectively inhibited by NFE2L3 (**Figure 5A**). However, and unexpectedly, NFE2L3 obviously elevated the ROS levels in MDA-MB-231 cells (**Figure 5B**). We supposed that excessive ROS in turn repressed the activation of downstream signaling pathways, as modulate levels of ROS is benefit for cancer cells while exorbitant accumulation of ROS is cytotoxic (35). More importantly, NFE2L3 not only increased drug resistance, but also inhibited the migration of MDA-MB-231 cells, whereas NFE2L3 silencing attenuated the function of miR-1246 inhibitor (**Figures 5C–G**).

Finally, the pro-oncogenic effect of miR-1246 was investigated using xenograft and metastasis mouse models. The present data revealed that the suppression of miR-1246 by a specific antagomir could effectively enhance the antitumor effect of Doc on xenograft growth by increasing cell apoptosis and likely by inhibiting NFE2L3 expression (**Figures 6A–E**). In addition, treatment with miR-1246 antagomir reduced the lung metastasis of breast cancer cells, as compared with those in the control (**Figure 6F**). Therefore, miR-1246 was shown to promote drug resistance and metastasis in breast cancer, *in vitro* as well as *in vivo*.

## Conclusion

In conclusion, the present study provided novel insight into the drug resistance and migration of human breast cancer cells *via* the miR-1246/NFE2L3 axis. Although the detailed underlying mechanisms must be further elucidated, it appears that NFE2L3 may play a key role in these processes. However, further studies are required to verify these findings.

## Data Availability Statement

The original contributions presented in the study are included in the article/supplementary material. Further inquiries can be directed to the corresponding author.

## Ethics Statement

The studies involving human participants were reviewed and approved by the Institutional Ethics Committee of the Taizhou Central Hospital (Zhejiang, China). The patients/participants provided their written informed consent to participate in this study.

## Author Contributions

Conceived and designed the experiments: J-GS and Y-CD. Performed the experiments: YiP, M-MQ, QC, YuP, Y-YR. Analyzed the data: J-GS, QC. Wrote the manuscript: J-GS and Y-CD. All authors contributed to the article and approved the submitted version.

## Funding

This work was supported by Technology Division of Taizhou (NO. 1801KY43), the Experiment Animal Science and Technology Project of Zhejiang Province (NO. 2016C37143), the Taizhou science and technology plan project (NO. 15yw07), and the Foundation of Taizhou University for Outstanding Young (2017YQ001).

## Conflict of Interest

The authors declare that the research was conducted in the absence of any commercial or financial relationships that could be construed as a potential conflict of interest.

## Publisher’s Note

All claims expressed in this article are solely those of the authors and do not necessarily represent those of their affiliated organizations, or those of the publisher, the editors and the reviewers. Any product that may be evaluated in this article, or claim that may be made by its manufacturer, is not guaranteed or endorsed by the publisher.

## References

[1] Siegel RL Miller KD. CA Cancer J Clin (2021) 71:7–33. doi: 10.3322/caac.21654 33433946

[2] Konieczkowski DJ Johannessen CM Garraway LA. A Convergence-Based Framework for Cancer Drug Resistance. Cancer Cell (2018) 33:801–15. doi: 10.1016/j.ccell.2018.03.025 PMC5957297 29763622

[3] Xie W Chu M Song G Zuo Z Han Z Chen C. Emerging Roles of Long Noncoding RNAs in Chemoresistance of Pancreatic Cancer. Semin Cancer Biol (2020) 15;S1044-579X(20)30222-4. doi: 10.1016/j.semcancer.2020.11.004 33207266

[4] Jin L Han B Siegel E Cui Y Giuliano A Cui X. Breast Cancer Lung Metastasis: Molecular Biology and Therapeutic Implications. Cancer Biol Ther (2018) 19:858–68. doi: 10.1080/15384047.2018.1456599 PMC6300341 29580128

[5] Qattan A. Novel miRNA Targets and Therapies in the Triple-Negative Breast Cancer Microenvironment: An Emerging Hope for a Challenging Disease. Int J Mol Sci (2020) 21(23):8905. doi: 10.3390/ijms21238905 PMC7727826 33255471

[6] Rupaimoole R Slack FJ. MicroRNA Therapeutics: Towards a New Era for the Management of Cancer and Other Diseases. Nat Rev Drug Discovery (2017) 16:203–22. doi: 10.1038/nrd.2016.246 28209991

[7] Yang F Luo LJ Zhang L Wang DD Yang SJ Ding L. MiR-346 Promotes the Biological Function of Breast Cancer Cells by Targeting SRCIN1 and Reduces Chemosensitivity to Docetaxel. Gene (2017) 600:21–8. doi: 10.1016/j.gene.2016.11.037 27913185

[8] Zhang X Zhong S Xu Y Yu D Ma T Chen L. MicroRNA-3646 Contributes to Docetaxel Resistance in Human Breast Cancer Cells by GSK-3beta/Beta-Catenin Signaling Pathway. PloS One (2016) 11:e0153194. doi: 10.1371/journal.pone.0153194 27045586 PMC4821636

[9] Tao WY Wang CY Sun YH Su YH Pang D Zhang GQ. MicroRNA-34c Suppresses Breast Cancer Migration and Invasion by Targeting Git1. J Cancer (2016) 7:1653–62. doi: 10.7150/jca.14762 PMC5039386 27698902

[10] Yan L Yu MC Gao GL Liang HW Zhou XY Zhu ZT. MiR-125a-5p Functions as a Tumour Suppressor in Breast Cancer by Downregulating BAP1. J Cell Biochem (2018) 119(11):8773–83. doi: 10.1002/jcb.27124 30076753

[11] Fu L Li Z Zhu J Wang P Fan G Dai Y. Serum Expression Levels of microRNA-382-3p, -598-3p, -1246 and -184 in Breast Cancer Patients. Oncol Lett (2016) 12:269–74. doi: 10.3892/ol.2016.4582 PMC4906595 27347136

[12] Guo S Chen J Chen F Zeng Q Liu WL Zhang G. Exosomes Derived From Fusobacterium Nucleatum-Infected Colorectal Cancer Cells Facilitate Tumour Metastasis by Selectively Carrying miR-1246/92b-3p/27a-3p and CXCL16. (2020) gutjnl-2020-321187. doi: 10.1136/gutjnl-2020-321187 33172926

[13] Huang YJ Huang TH Yadav VK Sumitra MR Tzeng DT Wei PL. Erratum: Preclinical Investigation of Ovatodiolide as a Potential Inhibitor of Colon Cancer Stem Cells *via* Downregulating Sphere-Derived Exosomal β-Catenin/STAT3/miR-1246 Cargoes. Am J Cancer Res (2020) 10:4640–2. PMC7783745 33415024

[14] Yang F Xiong H Duan L Li Q Li X Zhou Y. MiR-1246 Promotes Metastasis and Invasion of A549 Cells by Targeting GSK-3β-Mediated Wnt/β-Catenin Pathway. Cancer Res Treat (2019) 51:1420–9. doi: 10.4143/crt.2018.638 PMC6790833 30913872

[15] Zimmerman M Hu X Liu K. Experimental Metastasis and CTL Adoptive Transfer Immunotherapy Mouse Model. J Vis Exp (2010) 26(45):2077. doi: 10.3791/2077 PMC3159593 21178954

[16] Chai S Ng KY Tong M Lau EY Lee TK Chan KW. Octamer 4/microRNA-1246 Signaling Axis Drives Wnt/beta-Catenin Activation in Liver Cancer Stem Cells. Hepatology (2016) 64:2062–76. doi: 10.1002/hep.28821 27639189

[17] Sakha S Muramatsu T Ueda K Inazawa J. Exosomal microRNA miR-1246 Induces Cell Motility and Invasion Through the Regulation of DENND2D in Oral Squamous Cell Carcinoma. Sci Rep (2016) 6:38750. doi: 10.1038/srep38750 27929118 PMC5144099

[18] Bott A Erdem N Lerrer S Hotz-Wagenblatt A Breunig C Abnaof K. miRNA-1246 Induces Pro-Inflammatory Responses in Mesenchymal Stem/Stromal Cells by Regulating PKA and PP2A. Oncotarget (2017) 8:43897–914. doi: 10.18632/oncotarget.14915 PMC5546423 28159925

[19] Dvinge H Git A Graf S Salmon-Divon M Curtis C Sottoriva A. The Shaping and Functional Consequences of the microRNA Landscape in Breast Cancer. Nature (2013) 497:378–82. doi: 10.1038/nature12108 23644459

[20] Elebro K Borgquist S Simonsson M Markkula A Jirstrom K Ingvar C. Combined Androgen and Estrogen Receptor Status in Breast Cancer: Treatment Prediction and Prognosis in a Population-Based Prospective Cohort. Clin Cancer Res (2015) 21:3640–50. doi: 10.1158/1078-0432.CCR-14-2564 25904752

[21] Heerboth S Housman G Leary M Longacre M Byler S Lapinska K. EMT and Tumor Metastasis. Clin Transl Med (2015) 4:6. doi: 10.1186/s40169-015-0048-3 25852822 PMC4385028

[22] Thiery JP Acloque H Huang RY Nieto MA. Epithelial-Mesenchymal Transitions in Development and Disease. Cell (2009) 139:871–90. doi: 10.1016/j.cell.2009.11.007 19945376

[23] Sun CW Donze D Farmer S Ciavatta DJ Townes TM. Cloning and Functional-Characterization of Lcr-F1 - A Bzip Transcription Factor That Activates Erythroid-Specific, Human Globin Gene-Expression. Blood (1994) 84:A218–8. doi: 10.1093/nar/22.12.2383 PMC523699 8036168

[24] Nguyen T Nioi P Pickett CB. The Nrf2-Antioxidant Response Element Signaling Pathway and Its Activation by Oxidative Stress. J Biol Chem (2009) 284:13291–5. doi: 10.1074/jbc.R900010200 PMC2679427 19182219

[25] Giannoni E Parri M Chiarugi P. EMT and Oxidative Stress: A Bidirectional Interplay Affecting Tumor Malignancy. Antioxid Redox Signal (2012) 16:1248–63. doi: 10.1089/ars.2011.4280 21929373

[26] Talmadge JE Fidler IJ. AACR Centennial Series: The Biology of Cancer Metastasis: Historical Perspective. Cancer Res (2010) 70:5649–69. doi: 10.1158/0008-5472.CAN-10-1040 PMC4037932 20610625

[27] Holohan C Van Schaeybroeck S Longley DB Johnston PG. Cancer Drug Resistance: An Evolving Paradigm. Nat Rev Cancer (2013) 13:714–26. doi: 10.1038/nrc3599 24060863

[28] Abbaspour Babaei M Zaman Huri H Kamalidehghan B Yeap SK Ahmadipour F. Apoptotic Induction and Inhibition of NF-κb Signaling Pathway in Human Prostatic Cancer PC3 Cells by Natural Compound 2,2'-Oxybis (4-Allyl-1-Methoxybenzene), Biseugenol B, From Litsea Costalis: An *In Vitro* Study. Onco Targets Ther (2017) 10:277–94. doi: 10.2147/OTT.S102894 PMC5237594 28138251

[29] Andrews NC Kotkow KJ Ney PA Erdjument-Bromage H Tempst P Orkin SH. The Ubiquitous Subunit of Erythroid Transcription Factor NF-E2 Is a Small Basic-Leucine Zipper Protein Related to the V-Maf Oncogene. Proc Natl Acad Sci USA (1993) 90:11488–92. doi: 10.1073/pnas.90.24.11488 PMC48009 8265578

[30] Katsuoka F Yamamoto M. Small Maf Proteins (MafF, MafG, MafK): History, Structure and Function. Gene (2016) 586:197–205. doi: 10.1016/j.gene.2016.03.058 27058431 PMC4911266

[31] Sykiotis GP Bohmann D. Stress-Activated Cap'n'collar Transcription Factors in Aging and Human Disease. Sci Signal (2010) 3:re3. doi: 10.1126/scisignal.3112re3 20215646 PMC2991085

[32] Chevillard G Paquet M Blank V. Nfe2l3 (Nrf3) Deficiency Predisposes Mice to T-Cell Lymphoblastic Lymphoma. Blood (2011) 117:2005–8. doi: 10.1182/blood-2010-02-271460 21148084

[33] Chevillard G Nouhi Z Anna D Paquet M Blank V. Nrf3-Deficient Mice Are Not Protected Against Acute Lung and Adipose Tissue Damages Induced by Butylated Hydroxytoluene. FEBS Lett (2010) 584:923–8. doi: 10.1016/j.febslet.2010.01.028 20085761

[34] Xiao Q Pepe AE Wang G Luo Z Zhang L Zeng L. Nrf3-Pla2g7 Interaction Plays an Essential Role in Smooth Muscle Differentiation From Stem Cells. Arterioscler Thromb Vasc Biol (2012) 32:730–44. doi: 10.1161/ATVBAHA.111.243188 22247257

[35] Wen C Wang H Wu X He L Zhou Q Wang F. ROS-Mediated Inactivation of the PI3K/AKT Pathway Is Involved in the Antigastric Cancer Effects of Thioredoxin Reductase-1 Inhibitor Chaetocin. Cell Death Dis (2019) 10:809. doi: 10.1038/s41419-019-2035-x 31649256 PMC6813365

